# Synthesis, biological evaluation and in-silico ADME studies of novel series of thiazolidin-2,4-dione derivatives as antimicrobial, antioxidant and anticancer agents

**DOI:** 10.1186/s13065-022-00861-7

**Published:** 2022-09-15

**Authors:** Harsh Kumar, Davinder Kumar, Pradeep Kumar, Suresh Thareja, Minakshi Gupta Marwaha, Umashanker Navik, Rakesh Kumar Marwaha

**Affiliations:** 1 Department of Pharmaceutical Sciences, Maharshi Dayanand University, Rohtak, 124001 Haryana India; 2 Department of Pharmaceutical Sciences and Natural Products, Central University of Punjab, Ghudda, Bathinda, 151401 Punjab India; 3 Department of Pharmaceutical Sciences, Sat Priya College of Pharmacy, Rohtak, 124001 Haryana India; 4 Department of Pharmacology, Central University of Punjab, Ghudda, Bathinda, 151401 Punjab India

**Keywords:** Synthesis, Antimicrobial, Anticancer, Antioxidant, Characterization, ADME

## Abstract

**Background:**

A novel series of thiazolidine-2,4-dione molecules was derived and their chemical structures were established using physiochemical parameters and spectral techniques (^1^H-NMR, IR, MS etc.). The synthesized molecule were then evaluated for their antioxidant, anticancer and antimicrobial potential.

**Results and discussion:**

Serial tube dilution method was employed to evaluate the antimicrobial potential against selected fungal and bacterial strains by taking fluconazole and cefadroxil as reference antifungal and antibacterial drugs respectively. 2,2-diphenyl-1-picrylhydrazyl (DPPH) free radical scavenging activity was used to assess the antioxidant potential of the synthesized analogues. Further, the anticancer potential of the selected molecules was assessed against DU-145 cancer cell lines using MTT assay. The drug-likeness was also evaluated by studying in-silico ADME parameters of the synthesized analogues.

**Conclusion:**

In antioxidant evaluation studies, the analogue **H5** with IC_50_ = 14.85 μg/mL was found to be the most active molecule. The antimicrobial evaluation outcomes suggested that the molecules **H5**, **H13**, **H15** and **H18** possessed moderate to promising activity against the selected species of microbial strains having MIC range 7.3 µM to 26.3 µM. The results of anticancer evaluation revealed that all the screened derivatives possess mild anticancer potential. The *in-silico* ADME studies revealed that all the compounds were found to be drug-like.

## Introduction

The World Health Organization (WHO) reports indicate that the microbial drug resistance (MDR) caused due to continuous use of presently available antibiotics and the development of resistance against presently available anticancer drugs is the major concern for human life worldwide now a days [1, 2]. The clinical effectiveness of currently using antibiotics against most of the MDR strains, viz. vancomycin-resistant enterococci (VRE), multidrug-resistant staphylococcus aureus (MRSA) etc. is shrinking constantly [3, 4]. This prompts the medicinal chemist/pharmacologist to explore their research to find the alternative antimicrobial drug therapies [5].

Cancer, is one of the most terrible diseases and is the leading cause of death worldwide which accounts for approximately 17% of the total causalities. It has been charterized by uncontrolled and abnormal cell growth [6]. Inhibition of DNA replication and transcription to restrain the growth of tumor cells by currently used chemotherapeutic drugs is highly toxic [7], hence there is no ideal therapy currently available to cure cancer. This prompts medicinal chemists and researchers to find newer compounds having good antimicrobial and anticancer potential [2] with an innovative mode of action and lesser toxic effects.

Thiazolidine-2,4-dione (TZD), a highly bioactive five membered heterocyclic ring system containing nitrogen and sulfur atoms along with two carbonyl groups, has generated special interest among the scientific community not only due to its diverse pharmacological potential but also due to various possibilities of chemical modification [8, 9]. TZDs which are primarily known for their antidiabetic potential [10–12] have also exhibited diverse therapeutic activities such as analgesic, anti-inflammatory [13–15], wound healing [16], antiproliferative [17, 18], antimalarial [19], antitubercular [20, 21], hypolipidemic [22], antiviral [23], antimicrobial [24–26] and antioxidant [27, 28] etc.

Due to extreme surge in the cost of discovering new drug candidates, the drug discovery strategy has shifted to assess the comprehensive drug properties of the molecules under study at the earliest, for the clinical success of the drug candidates [29]. Pharmacokinetic parameters like absorption, distribution, metabolism and excretion (ADME) play a vital role in dose defining, overall safety margins and dose intervals during the drug development process [30]. Optimization of these parameters for a new chemical moiety having specific biological potential to be orally active is extremely important [5]. The molecules with poor ADME parameters may show unexpected toxicity, leading to withdrawal from the market and hence causing large financial losses [31].

Based on the literature survey and in continuation with our previous research efforts; in the present study, some new (4-oxothiazolidin-2-yl)thiazolidine-2,4-dione derivatives were synthesized and screened for antimicrobial, anticancer and antioxidant potential along with *in-silico* evaluation of ADME parameters of synthesized molecules.

## Results and discussion

### Chemistry

**Scheme 1:**
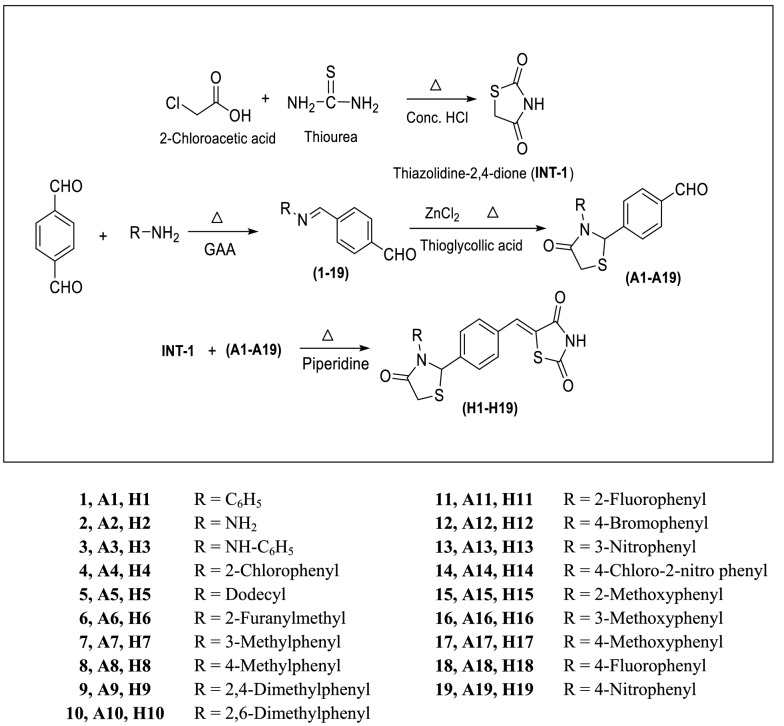
Synthesis of 5-(-4-(3-(substituted aryl/alkyl)-4-oxothiazolidin-2-yl)benzylidene) thiazolidine-2,4-dione derivatives (**H1–H19**)

TZD derivatives (**H1-H19**) were synthesized by employing a synthetic procedure as shown in Scheme 1. Initially, 2-chloroacetic acid was allowed to react with thiourea in presence of concentrated HCl to get TZD (**INT-I**). Schiff’s bases (**1–19**) were obtained by treating terephthalaldehyde and various substituted amines/anilines by taking acetic acid (glacial) as catalyst. Further compounds **1–19** were treated with thioglycolic acid using a small quantity of zinc chloride as catalyst to obtain intermediates 4-(4-oxo-3-substitutedaryl/alkylthiazolidin-2-yl)benzaldehyde (**A1–A19**). Finally the reaction of **INT-I** with intermediates **A1–A19** yielded final derivatives 5-(-4-(3-(substituted aryl/alkyl)-4-oxothiazolidin-2-yl)benzylidene)thiazolidine-2,4-dione (**H1–H19**). The physicochemical parameters and spectral analysis of the synthesized analogues are summarized in Table 1. The molecular structures of the derived analogues (**H1–H19**) were established using different spectral techniques viz. FT-IR (KBr, cm^−1^), ^1^H-NMR (DMSO-d_6,_ 400 MHz, δ ppm), Mass spectra and elemental analysis. The presence of stretching bands at 3029–2885 cm^−1^, 3199-2972 cm^−1^, 3498–3335 cm^−1^, 1800–1687 cm^−1^, 1556–1497 cm^−1^, in IR spectrum, indicated the presence of C–H (aliphatic), C–H (aromatic), N–H, C=O, C=C (aromatic), respectively in the derived analogues. The presence of absorption bands around 1381–1232 cm^−1^, 1696–1546 cm^−1^, and 1196–1038 cm^−1^ in IR spectrum, corresponded to stretching vibrations of C–N, C=C (aliphatic) and C–C, respectively. The presence of absorption bands in the range of 847–801 cm^−1^ corresponds to C–H out of plane bending vibrations in the molecules. Appearance of the bending absorption band at 680–618 cm^−1^ confirms the presence of C–S group. Compound **H4** possessed a stretching absorption band of C–Cl around 761 cm^−1^. Compounds **H12**, **H13** and **H19** displayed absorption bands at 1220–1176 cm^−1^ and 1437–1388 cm^−1^ of N–O and N=O groups respectively. The stretching band of O-CH_3_ group in compounds **H15**, **H16** and **H17** was seen at 1033–1025 cm^−1^. The aromatic protons present in the derived analogues were confirmed by the presence of multiplet signals between 6.68 and 7.95 δ ppm in the ^1^H-NMR spectra. The appearance of singlet(s) between 12.46–12.61 δ ppm, 7.49–7.97 δ ppm, 5.22–6.95 δ ppm confirmed the presence of –NH, –CH= and –CH of thiazolidin-4-one groups, respectively. In compound **H2** the presence of H of -NH_2_ group was confirmed by the presence of singlet (s) at 3.79 δ ppm. The presence of OCH_3_ of Ar–OCH_3_ in the compounds, **H15**, **H16** and **H17** was confirmed by the appearance of singlet(s) at 2.88–3.79 δ ppm. The presence of CH_3_ (Ar-CH_3_) in compounds **H8, H9** and **H10** was confirmed by the presence of singlet (s) at 2.12–2.73 δ ppm in ^1^H-NMR spectra. In compound **H3** appearance of singlet at 9.61 δ ppm confirmed the presence of NH of Ar–NH. The existence of dodecyl group in compound **H5** was confirmed by the appearance of triplet at 0.90 δ ppm of CH_3_, multiplet at 1.23–2.88 δ ppm of CH_2_ and multiplet at 3.711 δ ppm of CH_2_ adjacent to CH = N. The presence of furfuryl group in compound **H6** was confirmed due to the presence of a doublet signal at 7.12 δ ppm corresponding to –CH of furan ring at 3rd position, singlet signal at 4.79 δ ppm due to –CH_2_ group adjacent to furan ring, a triplet signal at 7.21 δ ppm due to –CH of furan ring at 4th position along with a doublet signal at 8.19 δ ppm due to –CH of furan ring adjacent to O (oxygen). The mass spectra of the derived analogues exhibited M^+^, M^+^ + 1 and M^+ ^− 1 peak.

**Table 1:** The physicochemical, elemental and spectral characteristics of synthesized thiazolidine-2,4-dione derivatives

Compound	Physicochemical and spectral characteristics
	(E)-5-(4-(4-oxo-3-phenylthiazolidin-2-yl)benzylidene)thiazolidin-2,4-dione: m.p. ^o^C: 280–282; Rf value: 0.56^*^; % yield: 74; IR (KBr pellets) cm^−1^: 3448.73 (N–H str.), 1770.59 (C=O str.), 1497.42 (C=C str., aromatic ring), 1661.76 (C=C str., aliphatic), 2943.38 (C–H str., aliphatic), 1255.61 (C–N str.), 847.82 (C–H oop bend., aromatic), 663.15 (C–S bend.) 1101.06 (C–C str., aromatic ring); ^1^H-NMR (δ, DMSO): 7.10–7.88 (m, 9H, Ar–H), 7.94 (s, 1H, –CH=), 3.44, 3.46 (d, 2H, –CH_2_ of thiazolidine-4-one), 6.77 (s, 1H, –CH of thiazolidine-4-one) 12.59 (s, 1H, NH); M. Formula: C_19_H_14_N_2_O_3_S_2;_ MS: m/z 383.04 (M^+^); Elemental analysis (CHN) Theoretical calc: C, 59.67; H, 3.69; N, 7.32 Found: C, 59.7; H, 3.67; N, 7.30
	(E)-5-(4-(3-amino-4-oxothiazolidin-2-yl)benzylidene)thiazolidin-2,4-dione: m.p. ^o^C: 208–210; Rf value: 0.61^**^; % yield: 60; IR (KBr pellets) cm^−1^: 3472.36 (N–H str.), 1690.31 (C=O str.), 1524.13 (C = C str., aromatic ring), 1637.59 (C=C str., aliphatic), 3119.42 (C–H str., aromatic ring), 3003.75 (C–H str., aliphatic), 1336.47 (C–N str.), 801.42 (C–H oop bend., aromatic), 628.31 (C–S bend.) 1149.31 (C–C str., aromatic ring) 3313.97, 3382.58 (N–H str., aliphatic amine); ^1^H-NMR (δ, DMSO): 6.98–7.78 (m, 4H, Ar–H), 7.95 (s, 1H, –CH=), 3.45, 3.58 (d, 2H, –CH_2_ of thiazolidine-4-one), 5.22 (s, 1H, –CH of thiazolidine-4-one), 3.79 (s, 2H, –NH_2_ group), 12.61 (s, 1H, NH); M. Formula: C_13_H_11_N_3_O_3_S_2_; MS: m/z 323.21 (M^+^ + 2); Elemental analysis (CHN) Theoretical calc: C, 48.59; H, 3.45; N, 13.08 Found: C, 48.62; H, 3.44; N, 13.10
	(E)-5-(4-(4-oxo-3-(phenylamino)thiazolidin-2-yl)benzylidene)thiazolidin-2,4-dione: m.p. ^o^C: 282–284; Rf value: 0.51^**^; % yield: 65; IR (KBr pellets) cm^−1^: 3477.42 (N–H str.), 3357.17 (N–H str., secondary amine), 1694.69 (C=O str.), 1518.27 (C=C str., aromatic ring), 1649.21 (C=C str., aliphatic), 3191.78 (C–H str., aromatic ring), 2950.88 (C–H str., aliphatic), 1274.86 (C–N str.), 801.86 (C–H oop bend., aromatic), 631.19 (C–S bend.), 1166.66 (C–C str., aromatic ring); ^1^H-NMR (δ, DMSO): 6.68–7.506 (m, 9H, Ar–H), 7.61 (s, 1H, –CH=), 3.32, 3.34 (d, 2H, –CH_2_ of thiazolidine-4-one), 5.71 (s, 1H, –CH of thiazolidine-4-one), 9.61 (s, 1H, NH adjacent to phenyl ring), 12.51 (s, 1H, NH of thiazolidine ring); M. Formula: C_19_H_15_N_3_O_3_S_2_; MS: m/z 398.27 (M^+^ + 1); Elemental analysis (CHN) Theoretical calc: C, 57.42; H, 3.80; N, 10.57 Found: C, 57.40; H, 3.79; N, 10.55
	(E)-5-(4-(3-(2-chlorophenyl)-4-oxothiazolidin-2-yl)benzylidene)thiazolidin-2,4-dione: m.p. ^o^C: 222–224; Rf value: 0.63^*^; % yield: 62; IR (KBr pellets) cm^−1^: 3335.73 (N–H str.), 1743.82 (C=O str.), 1520.84 (C=C str., aromatic ring), 1693.17 (C=C str., aliphatic), 3186.47 (C–H str., aromatic ring), 2981.68 (C–H str., aliphatic), 1293.41 (C–N str.), 836.53 (C–H oop bend., aromatic), 623.85 (C–S bend.), 761.83 (C–Cl bend., o-substitution on phenyl ring) 1069.19 (C–C str., aromatic ring); M. Formula: C_19_H_13_ClN_2_O_3_S_2_; MS: m/z 415.47 (M^+^ − 1); Elemental analysis (CHN) Theoretical calc: C, 54.74; H, 3.14; N, 6.72 Found: C, 54.76; H, 3.15; N, 6.70
	(E)-5-(4-(3-dodecyl-4-oxothiazolidin-2-yl)benzylidene)thiazolidin-2,4-dione: m.p. ^o^C: 160–162; Rf value: 0.57^**^; % yield: 70; IR (KBr pellets) cm^−1^: 3393.96 (N–H str.), 1692.08 (C=O str.), 1515.19 (C=C str., aromatic ring), 1612.17 (C=C str., aliphatic), 3160.90 (C–H str., aromatic ring), 3020.88 (C–H str., aliphatic), 1296.92 (C–N str.), 841.16 (C–H oop bend., aromatic), 661.02 (C–S bend.), 1067.74 (C–C str., aromatic ring) 2915.30 (C–H str., side chain); ^1^H NMR (δ, DMSO): 7.04–7.65 (m, 4H, Ar–H), 7.95 (s, 1H, –CH=), 3.87, 3.99 (d, 2H, –CH_2_ of thiazolidine-4-one), 4.90 (s, 1H, –CH of thiazolidine-4-one), 12.53 (s, 1H, NH), 1.23–2.88 (m, 20H, CH_2_), 0.90 (t, J=85 Hz, 3H, CH_3_), 3.71 (m, 2H, CH_2_ adjacent to CH=N); M. Formula: C_25_H_34_N_2_O_3_S_2;_ MS: m/z 476.1 (M^+^-1); Elemental analysis (CHN) Theoretical calc: C, 63.26; H, 7.22; N, 5.90 Found: C, 63.25; H, 7.24; N, 5.91
	(E)-5-(4-(3-(furan-2-ylmethyl)-4-oxothiazolidin-2-yl)benzylidene)thiazolidin-2,4-dione: m.p. ^o^C: 127–129; Rf value: 0.56^*^; % yield: 60; IR (KBr pellets) cm^−1^: 3437.28 (N–H str.), 1760.34 (C=O str.), 1549.27 (C=C str., aromatic ring), 1660.96 (C=C str., aliphatic), 3199.60 (C–H str., aromatic ring), 2901.27 (C–H str., aliphatic), 1339.28 (C–N str.), 844.33 (C–H oop bend., aromatic), 660.47 (C–S bend.), 1096.59 (C–C str.), 1018.24 (C–O–C str., furan ring); ^1^H NMR (δ, DMSO): 7.12–7.95 (m, 4H, Ar–H), 7.97 (s, 1H, –CH=), 3.76, 3.97 (d, 2H, –CH_2_ of thiazolidine-4-one), 6.59 (s, 1H, –CH of thiazolidine-4-one), 12.58 (s, 1H, NH), 4.79 (s, 2H, –CH_2_ adjacent to furan ring), 7.21 (d, 1H, CH of furan ring at 3rd position), 7.12 (t, 1H, CH of furan ring at 4th position), 8.17 (d, 1H, CH of furan ring adjacent to O); M. Formula: C_17_H_12_N_2_O_4_S_2;_ Elemental analysis (CHN) Theoretical calc: C, 54.83; H, 3.25; N, 7.52 Found: C, 54.85; H, 3.22; N, 7.53
	(E)-5-(4-(4-oxo-3-(m-tolyl)thiazolidin-2-yl)benzylidene)thiazolidin-2,4-dione: m.p. ^o^C: 141–143; Rf value: 0.61^**^; % yield: 61; IR (KBr pellets) cm^−1^: 3423.62 (N–H str.), 1694.32 (C=O str.), 1514.80 (C=C str., aromatic ring), 1645.13 (C=C str., aliphatic), 3117.59 (C–H str., aromatic ring), 2966.25 (C–H str., aliphatic), 1335.01 (C–N str.), 824.35 (C–H oop bend., aromatic), 680.72 (C–S bend.), 1108.60 (C–C str.) 2873.73 (C–H str., side chain); M. Formula: C_20_H_16_N_2_O_3_S_2;_ Elemental analysis (CHN) Theoretical calc: C, 60.59; H, 4.07; N, 7.07 Found: C, 60.60; H, 4.06; N, 7.07
	(E)-5-(4-(4-oxo-3-(p-tolyl)thiazolidin-2-yl)benzylidene)thiazolidin-2,4-dione: m.p. ^o^C: 191–193; Rf value: 0.54^*^; % yield: 61; IR (KBr pellets) cm^−1^: 3471.71 (N–H str.), 1689.58 (C=O str.), 1538.27 (C=C str., aromatic ring), 1646.90 (C=C str., aliphatic), 3163.62 (C–H str., aromatic ring), 3022.64 (C–H str., aliphatic), 1328.53 (C–N str.), 809.91 (C–H oop bend., aromatic), 679.58 (C–S bend.), 1140.25 (C–C str. aromatic ring) 2946.07 (C–H str., side chain); ^1^H NMR (δ, DMSO): 7.03–7.75 (m, 8H, Ar–H), 7.94 (s, 1H, –CH=), 3.76, 3.97 (d, 2H, –CH_2_ of thiazolidine-4-one), 6.46 (s, 1H, –CH of thiazolidine-4-one), 12.55 (s, 1H, NH), 2.73(s, 3H, CH_3,_ p-position); M. Formula: C_20_H_16_N_2_O_3_S_2_; Elemental analysis (CHN) Theoretical calc: C, 60.59; H, 4.07; N, 7.07 Found: C, 60.62; H, 4.09; N, 7.05
	(E)-5-(4-(3-(2,4-dimethylphenyl)-4-oxothiazolidin-2-yl)benzylidene)thiazolidin-2,4-dione: m.p. ^o^C: 231–233; Rf value: 0.59^**^; % yield: 60; IR (KBr cm^−1^): 3498.49 (N–H str.), 1743.71 (C=O str.), 1542.27 (C=C str., aromatic ring), 1696.07 (C=C str., aliphatic), 3178.82 (C–H str., aromatic ring), 3029.83 (C–H str., aliphatic), 1268.36 (C–N str.), 838.86 (C–H oop bend., aromatic), 661.91 (C–S bend., thiazolidine ring), 1038.69 (C–C str.) 2978.93 (C–H str., side chain); ^1^H NMR (δ, DMSO): 7.20–7.42 (m, 7H, Ar–H), 7.49 (s, 1H, –CH=), 3.68, 3.75 (d, 2H, –CH_2_ of thiazolidine-4-one), 6.48 (s, 1H, –CH of thiazolidine-4-one), 12.61 (s, 1H, NH), 2.23(s, 3H, CH_3,_ p-position), 2.16 (s, 3H, CH_3_ o-position); M. Formula: C_21_H_19_N_2_O_3_S_2;_ Elemental analysis (CHN) Theoretical calc: C, 61.44; H, 4.22; N, 6.82 Found: C, 61.45; H, 4.23; N, 6.81
	(E)-5-(4-(3-(2,6-dimethylphenyl)-4-oxothiazolidin-2-yl)benzylidene)thiazolidin-2,4-dione: m.p. ^o^C: 145–147; Rf value: 0.63^**^; % yield: 56; IR (KBr cm^−1^): 3475.21 (N–H str.), 1687.76 (C=O str.), 1508.71 (C=C str., aromatic ring), 1546.87 (C=C str., aliphatic), 2997.90 (C–H str., aromatic ring), 2948.20 (C–H str., aliphatic), 1232.86 (C–N str., thiazolidine ring), 629.87 (C–S bend.), 1094.67 (C–C str.) 2821.33 (C–H str., side chain); ^1^H NMR (δ, DMSO): 7.15–7.30 (m, 7H, Ar–H), 7.95 (s, 1H, –CH =), 3.93, 4.05 (d, 2H, –CH_2_ of thiazolidine-4-one), 6.52 (s, 1H, –CH of thiazolidine-4-one), 12.49 (s, 1H, NH), 2.12 (s, 6H, CH_3_ o-position); M. Formula: C_21_H_19_N_2_O_3_S_2;_ Elemental analysis (CHN) Theoretical calc: C, 61.44; H, 4.22; N, 6.82 Found: C, 61.42; H, 4.21; N, 6.83
	(E)-5-(4-(3-(2-fluorophenyl)-4-oxothiazolidin-2-yl)benzylidene)thiazolidin-2,4-dione: m.p. ^o^C: 121–123; Rf value: 0.53^*^; % yield: 69; IR (KBr cm^−1^): 3397.62 (N–H str.), 1690.56 (C=O str.), 1556.85 (C=C str., aromatic ring), 1621.56 (C=C str., aliphatic), 3022.29 (C–H str., aromatic ring), 2942.43 (C–H str., aliphatic), 1308.11 (C–N str.), 831.66 (C–H oop bend., aromatic), 666.00 (C–S bend.), 1196.21 (C–C str.), 1016.77 (C-F bend., o-substitution on phenyl ring); ^1^H NMR (δ, DMSO): 7.19–7.21 (m, 8H, Ar–H), 7.951 (s, 1H, –CH=), 3.694, 3.961 (d, 2H, –CH_2_ of thiazolidine-4-one), 6.39 (s, 1H, –CH of thiazolidine-4-one), 12.57 (s, 1H, NH); M. Formula: C_19_H_13_FN_2_O_3_S_2_; MS: m/z 401.03 (M^+^ + 1); Elemental analysis (CHN) Theoretical calc: C, 56.99; H, 3.27; N, 7.00 Found: C, 57.00; H, 3.25; N, 7.01
	(E)-5-(4-(3-(4-bromophenyl)-4-oxothiazolidin-2-yl)benzylidene)thiazolidin-2,4-dione: m.p. ^o^C: 119–121; Rf value: 0.59^**^; % yield: 73; IR (KBr cm^−1^): 3381.80 (N–H str.), 1746.01 (C=O str.), 1512.02 (C=C str., aromatic ring), 1672.91 (C=C str., aliphatic), 3110.07 (C–H str., aromatic ring), 2966.14 (C–H str., aliphatic), 1243.62 (C–N str.), 1156.04 (C–C str.), 826.49 (C–H oop bend., aromatic), 680.92 (C–Br bend., p-substitution on phenyl ring); ^1^H NMR (δ, DMSO): 7.21–7.49 (m, 8H, Ar–H), 7.952 (s, 1H, –CH=), 3.691, 3.981 (d, 2H, –CH_2_ of thiazolidine-4-one), 6.51 (s, 1H, –CH of thiazolidine-4-one), 12.63 (s, 1H, NH); M. Formula: C_19_H_13_BrN_2_O_3_S_2_; MS: m/z 459.91 (M^+^-1); Elemental analysis (CHN) Theoretical calc: C, 49.41; H, 3.27; N, 7.00 Found: C, 49.48; H, 3.25; N, 7.01
	(E)-5-(4-(3-(3-nitrophenyl)-4-oxothiazolidin-2-yl)benzylidene)thiazolidin-2,4-dione: m.p. ^o^C: 159–161; Rf value: 0.58^*^; % yield: 74; IR (KBr cm^−1^): 3380.87 (N–H str.), 1717.77 (C=O str.), 1541.24 (C=C str., aromatic ring), 1663.52 (C=C str., aliphatic), 3007.25 (C–H str., aromatic ring), 2885.82 (C–H str., aliphatic), 1250.32 (C–N str.), 618.67 (C–S bend.), 1079.45 (C–C str.), 1220.79 (N–O str., m-substitution on phenyl ring), 1436.75 (N=O str., m-substitution on phenyl ring); ^1^H NMR (δ, DMSO): 7.211–7.523 (m, 8H, Ar–H), 7.950 (s, 1H, –CH=), 4.098, 4.132 (d, 2H, –CH_2_ of thiazolidine-4-one), 6.49 (s, 1H, –CH of thiazolidine-4-one), 12.61 (s, 1H, NH); M. Formula: C_19_H_13_N_3_O_5_S_2_; Elemental analysis (CHN) Theoretical calc: C, 53.39; H, 3.07; N, 9.83 Found: C, 53.41; H, 3.05; N, 9.85
	(E)-5-(4-(3-(4-chloro-2-nitrophenyl)-4-oxothiazolidin-2-yl)benzylidene)thiazolidin-2,4-dione: m.p. ^o^C: 117–119; Rf value: 0.51^*^; % yield: 67; IR (KBr cm^−1^): 1509.11 (C=C str., aromatic ring), 2972.95 (C–H str., aromatic ring), 2885.16 (C–H str., aliphatic), 1249.06 (C–N str.), 622.84 (C–S bend., thiazolidine ring), 1074.34 (C–C str.), 1176.56 (N–O str., o-substitution on phenyl ring), 1437.32 (N=O str., o-substitution on phenyl ring), 640.18 (C–Cl bend., p-substitution on phenyl ring); ^1^H NMR (δ, DMSO): 7.092–7.269 (m, 7H, Ar–H), 7.953 (s, 1H, –CH =), 3.76, 3.88 (d, 2H, –CH_2_ of thiazolidine-4-one), 6.53 (s, 1H, –CH of thiazolidine-4-one), 12.46 (s, 1H, NH), M. Formula: C_19_H_12_ClN_3_O_5_S_2_; MS: m/z 462.1 (M^+^); Elemental analysis (CHN) Theoretical calc: C, 49.41; H, 2.62; N, 9.10 Found: C, 49.43; H, 2.61; N, 9.11
	(E)-5-(4-(3-(2-methoxyphenyl)-4-oxothiazolidin-2-yl)benzylidene)thiazolidin-2,4-dione: m.p. ^o^C: 131–133; Rf value: 0.67^**^; % yield: 65; IR (KBr cm^−1^): 3449.87 (N–H str.), 1748.92 (C=O str.), 1536.40 (C=C str., aromatic ring), 1659.37 (C=C str., aliphatic), 3138.14 (C–H str., aromatic ring), 3011.17 (C–H str., aliphatic), 1323.04 (C–N str.), 835.87 (C–H oop bend., aromatic), 659.25 (C–S bend.), 1101.58 (C–C str.), 1033.96 (O–CH_3_ str., o-substitution on phenyl ring); ^1^H NMR (δ, DMSO): 7.063–7.595 (m, 8H, Ar–H), 7.949 (s, 1H, –CH=), 3.99, 4.02 (d, 2H, –CH_2_ of thiazolidine-4-one), 5.88 (s, 1H, –CH of thiazolidine-4-one), 12.53 (s, 1H, NH), 3.795 (s, 3H, OCH_3_, o-position); M. Formula: C_20_H_16_N_2_O_4_S_2_; MS: m/z 413.24 (M^+^ + 1); Elemental analysis (CHN) Theoretical calc: C, 58.24; H, 3.91; N, 6.79 Found: C, 58.25; H, 3.90; N, 6.80
	(E)-5-(4-(3-(3-methoxyphenyl)-4-oxothiazolidin-2-yl)benzylidene)thiazolidin-2,4-dione: m.p. ^o^C: 196–198; Rf value: 0.61^**^; % yield: 65; IR (KBr cm^−1^): 3438.44 (N–H str.), 1741.31 (C=O str.), 1543.46 (C=C str., aromatic ring), 1664.46 (C=C str., aliphatic), 2899.96 (C–H str., aliphatic), 3089.91 (C–H str., aromatic ring), 1381.07 (C–N str.), 822.62 (C–H oop bend., aromatic), 649.37 (C–S bend.), 1115.13 (C–C str.), 1025.29 (O-CH_3_ str., m-substitution on phenyl ring); ^1^H NMR (δ, DMSO): 7.208–7.393 (m, 8H, Ar–H), 7.95 (s, 1H, –CH=), 3.61, 3.92 (d, 2H, -CH_2_ of thiazolidine-4-one), 6.16 (s, 1H, –CH of thiazolidine-4-one), 12.50 (s, 1H, NH), 2.89 (s, 3H, OCH_3_, m-position); M. Formula: C_20_H_16_N_2_O_4_S_2_; MS: m/z 413.63 (M^+^ + 1); Elemental analysis (CHN) Theoretical calc: C, 58.24; H, 3.91; N, 6.79 Found: C, 58.23; H, 3.88; N, 6.78
	(E)-5-(4-(3-(4-methoxyphenyl)-4-oxothiazolidin-2-yl)benzylidene)thiazolidin-2,4-dione: m.p. ^o^C: 130–132; Rf value: 0.64^**^; % yield: 70; IR (KBr cm^−1^): 3361.69 (N–H str.), 1693.75 (C=O str.), 1509.86 (C=C str., aromatic ring), 1606.81 (C=C str., aliphatic), 2922.73 (C–H str., aliphatic), 1395.80 (C–N str.), 651.12 (C–S bend., thiazolidine ring), 1027.91 (O–CH_3_ str., o-substitution on phenyl ring); ^1^H NMR (δ, DMSO): 7.206–7.445 (m, 8H, Ar–H), 7.827 (s, 1H, –CH=), 3.86, 3.91 (d, 2H, –CH_2_ of thiazolidine-4-one), 6.951 (s, 1H, –CH of thiazolidine-4-one), 12.45 (s, 1H, NH), 3.33 (s, 3H, OCH_3,_ p-position); M. Formula: C_20_H_16_N_2_O_4_S_2_; MS: m/z 414.93 (M^+^ + 2); Elemental analysis (CHN) Theoretical calc: C, 58.24; H, 3.91; N, 6.79 Found: C, 58.21; H, 3.89; N, 6.77
	(E)-5-(4-(3-(4-fluorophenyl)-4-oxothiazolidin-2-yl)benzylidene)thiazolidin-2,4-dione: m.p. ^o^C: 150–152; Rf value: 0.57^*^; % yield: 60; IR (KBr cm^−1^): 3414.71 (N–H str.), 1800.02 (C=O str.), 1501.61 (C=C str., aromatic ring), 1660.40 (C=C str., aliphatic), 3064.39 (C–H str., aromatic ring), 2924.57 (C–H str., aliphatic), 1288.86 (C–N str., thiazolidine ring), 824.35 (C–H oop bend., aromatic), 660.68 (C–S bend., thiazolidine ring), 1095.34 (C–C str.), 1019.92 (C-F str., p-substitution on phenyl ring); ^1^H NMR (δ, DMSO): 7.12–7.69 (m, 8H, Ar–H), 7.951 (s, 1H, –CH=), 3.90, 3.98 (d, 2H, -CH_2_ of thiazolidine-4-one), 6.82 (s, 1H, –CH of thiazolidine-4-one), 12.51 (s, 1H, NH);M. Formula: C_19_H_13_FN_2_O_3_S_2_; MS: m/z 401.5 (M^+^ + 1); Elemental analysis (CHN) Theoretical calc: C, 56.99; H, 3.27; N, 7.00 Found: C, 56.97; H, 3.26; N, 7.03
	(E)-5-(4-(3-(4-nitrophenyl)-4-oxothiazolidin-2-yl)benzylidene)thiazolidin-2,4-dione: m.p. ^o^C: 144–146; Rf value: 0.60^*^; % yield: 70; IR (KBr cm^−1^): 3391.53 (N–H str.), 1788.47 (C=O str.), 1502.43 (C=C str., aromatic ring), 1660.13 (C=C str., aliphatic), 3115.33 (C–H str., aromatic ring), 2977.35 (C–H str., aliphatic), 1300.51 (C–N str.), 823.74 (C–H oop bend., aromatic), 663.28 (C–S bend.), 1097.38 (C–C str.), 1200.58 (N–O str., p-substitution on phenyl ring), 1388.68 (N=O str., p-substitution on phenyl ring); ^1^H NMR (δ, DMSO): 7.29–7.70 (m, 8H, Ar–H), 7.951 (s, 1H, -CH =), 3.90, 4.01 (d, 2H, -CH_2_ of thiazolidine-4-one), 6.52 (s, 1H, –CH of thiazolidine-4-one), 12.59 (s, 1H, NH); M. Formula: C_19_H_13_N_3_O_5_S_2_; Elemental analysis (CHN) Theoretical calc: C, 53.39; H, 3.03; N, 9.83 Found: C, 53.35; H, 3.05; N, 9.87[]

TLC mobile phase=^*^n-Hexane: Ethyl Acetate:: 1: 1, ^**^n-Hexane: Ethyl Acetate:: 3: 7

### Antimicrobial screening

**Fig. 1:**
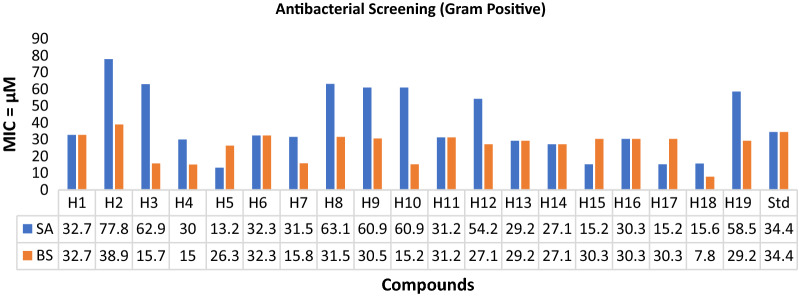
Antibacterial evaluation results against Gram positive species using cefadroxil as standard drug

**Fig. 2:**
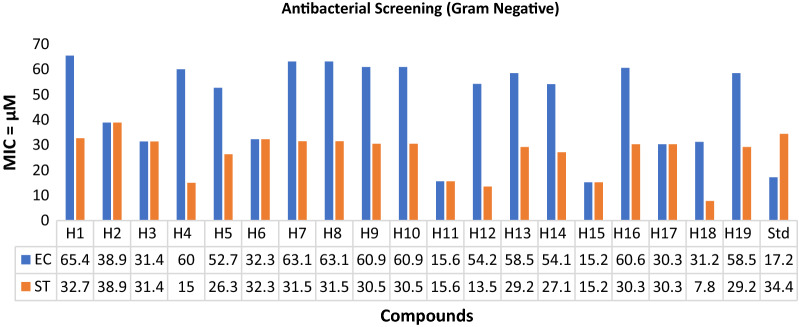
Antibacterial evaluation results against Gram negative species using cefadroxil as standard drug

**Fig. 3:**
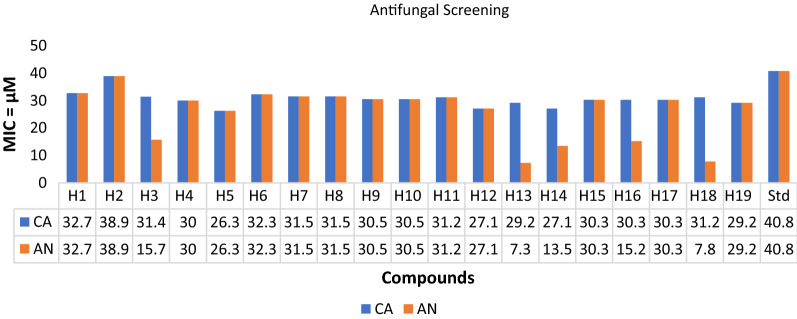
Antifungal evaluation results against fungal species using fluconazole as standard drug

The in vitro antimicrobial evaluation of the synthesized analogues was carried out using a serial tube dilution procedure [32] (Table 2; Figs. 1, 2, 3). The antifungal screening results indicated the compound **H5** to be moderately activity against *C. albicans* (MIC = 26.3 µM) and compound **H13** exhibited promising activity against *A. niger* (MIC = 7.3 µM), respectively. The results of antibacterial screening revealed that compound **H5** was moderately active against *S. aureus* (MIC = 13.2 µM). Antimicrobial screening results further revealed that compound **H18** possessed promising activity against *B. subtilis* and *S. typhi* (MIC = 7.8 µM each) whereas compound **H15** (MIC = 15.2 µM) has shown moderate activity against *E. coli* strain. The results of antifungal screening revealed that the derived analogues possess superior activity against both the selected strains of fungus i.e., *A. niger* and *C. albicans* while antibacterial screening results exhibited mild to moderate activity against the selected strains in comparison to cefadroxil as standard drug. So, these molecules can be viewed as lead structures for further development and optimization into potent antimicrobial agents.

**Table 2:** In vitro antimicrobial activity of the synthesized compounds

Comp.	Antimicrobial screening (MIC = µM)					
	**SA**	**BS**	**EC**	**ST**	**CA**	**AN**
**H1**	32.7	32.7	65.4	32.7	32.7	32.7
**H2**	77.8	38.9	38.9	38.9	38.9	38.9
**H3**	62.9	15.7	31.4	31.4	31.4	15.7
**H4**	30.0	15.0	60.0	15.0	30.0	30.0
**H5**	**13.2**	26.3	52.7	26.3	**26.3**	26.3
**H6**	32.3	32.3	32.3	32.3	32.3	32.3
**H7**	31.5	15.8	63.1	31.5	31.5	31.5
**H8**	63.1	31.5	63.1	31.5	31.5	31.5
**H9**	60.9	30.5	60.9	30.5	30.5	30.5
**H10**	60.9	15.2	60.9	30.5	30.5	30.5
**H11**	31.2	31.2	15.6	15.6	31.2	31.2
**H12**	54.2	27.1	54.2	13.5	27.1	27.1
**H13**	29.2	29.2	58.5	29.2	29.2	**7.3**
**H14**	27.1	27.1	54.1	27.1	27.1	13.5
**H15**	15.2	30.3	**15.2**	15.2	30.3	30.3
**H16**	30.3	30.3	60.6	30.3	30.3	15.2
**H17**	15.2	30.3	30.3	30.3	30.3	30.3
**H18**	15.6	**7.8**	31.2	**7.8**	31.2	7.8
**H19**	58.5	29.2	58.5	29.2	29.2	29.2
**Cefadroxil**	34.4	34.4	17.2	34.4	–	–
**Fluconazole**	–	–	–	–	40.8	40.8

SA: *Staphylococcus aureus*, BS: *Bacillus subtilis*, EC: *Escherichia coli*, ST: *Salmonella typhi*; CA: *Candida albicans*, AN: *Aspergillus niger*

### Antioxidant evaluation

**Fig. 4:**
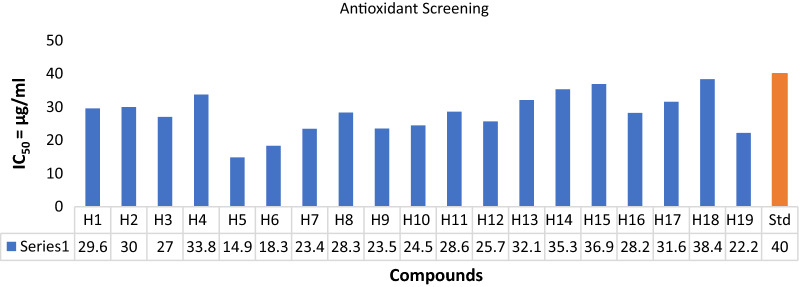
Antioxidant screening of synthesized compounds using ascorbic acid as standard drug

DPPH free radical scavenging assay was performed to assess the antioxidant potential of the newly synthesized derivatives using ascorbic acid as a reference drug [33]. DPPH assay is among the most utilized methods used for assessing the antioxidant potential of a compound which is based on a chain-breaking mechanism. DPPH is a stable free radical which can be transformed into a constant diamagnetic molecule by accepting hydrogen or an electron radical from the antioxidant compound [34]. The DPPH solution (methanolic) exhibit a strong absorption band at 517 nm. As DPPH radical reacts with the antioxidant/ reducing agent, a new bond is generated which leads to decreases in the color intensity of the solution. As the strength of antioxidants in the solution is increased, the DPPH radical takes up a greater number of electrons, leading to a loss in the color intensity of the solution from purple to pale yellow which is monitored spectrophotometrically at 517 nm [35]. The IC_50_ value (μg/mL) and % inhibition for all the synthesized molecules were calculated. The antioxidant screening assay revealed that the derived molecules were more potent than the reference drug itself. Further, the antioxidant screening showed compound **H5** (IC_50_ = 14.85 μg/mL) be the most potent. Antioxidant evaluation results are depicted in Table 3 and Fig. 4.

**Table 3:** In vitro antioxidant activity of the synthesized compounds

Comp.	% Inhibition				Antioxidant activity (IC_50_ = µg/mL)
	25 µg/mL	50 µg/mL	75 µg/mL	100 µg/mL	
**H1**	21.18	38.03	50.98	63.92	29.58
**H2**	36.86	43.92	49.80	56.47	29.99
**H3**	27.45	43.13	56.86	62.74	27.04
**H4**	15.68	36.47	45.88	56.07	33.78
**H5**	43.92	56.08	67.84	76.08	14.85
**H6**	40.78	52.94	61.57	72.16	18.32
**H7**	29.02	48.63	62.35	68.24	23.43
**H8**	21.18	40.78	55.69	63.53	28.31
**H9**	36.86	42.35	54.90	72.94	23.53
**H10**	27.84	48.24	60.39	66.27	24.46
**H11**	32.55	43.14	51.76	59.61	28.60
**H12**	23.53	47.84	57.25	67.45	25.69
**H13**	21.96	32.16	48.24	60.39	32.09
**H14**	9.41	21.96	40.00	47.45	35.31
**H15**	12.94	29.80	44.71	50.98	36.93
**H16**	32.16	41.96	48.24	64.31	28.24
**H17**	22.35	32.16	52.55	58.82	31.57
**H18**	9.41	26.27	36.86	52.55	38.37
**H19**	36.47	48.63	60.39	65.49	22.22
**Ascorbic Acid**	16.08	30.20	40.39	48.62	40

### ADME results

The synthesized derivatives (**H1–H19**) were submitted to QikProp module of Schrödinger software 2020–4 (Maestro version *12.5*) for the calculation of ADME parameters [36]. The results of ADME studies were promising and within the defined range of Qikprop module. Various physically relevant and pharmacologically significant parameters of the synthesized derivatives were studied and found to be within Lipinski’s rule of five range. The parameters studied included Predicted skin permeability (QPlogKp = − 8.0 to − 1.0), Molecular weight of the molecule (mol. MW ≤ 500), human oral absorption (1, 2 or 3), Predicted blood /brain partition coefficient (QPlogBB = − 3.0 to − 1.2), Predicted gas/water partition coefficient (QPlogPw = 4.0 to − 45.0), Predicted water/octanol partition coefficient (QPlogPo/w = − 2.0 to − 6.5), Percent human oral absorption (0–100), accept HB (2.0 to − 20.0), donor HB (0.0 to − 6.0) and results revealed these molecules as appropriate drug candidates. The ADME studies results are summarized in Table 4.

**Table 4:** *In-silico* ADME parameters of synthesized compounds

Comp	ADME parameters										
	Mol MW	Rule of Five	QPlogPo/w	Human Oral Absorption	Volume	% Human Oral Absorption	QPlogP_w_	QPlogK_p_	QPlogBB	Donor HB	Accept HB
**H1**	382.451	0	2.938	3	1083.522	88.762	11.638	− 3.26	− 0.864	1.0	6.5
**H2**	321.318	0	1.017	3	895.076	67.663	13.451	− 4.786	− 1.339	3.0	6.5
**H3**	397.466	0	2.648	3	1071.33	88.868	12.756	− 2.99	− 0.716	2.0	7.0
**H4**	416.896	0	3.378	3	1121.417	91.86	11.479	− 3.3	− 0.0.685	1.0	6.5
**H5**	474.675	1	5.179	1	1560.744	86.143	11.023	− 2.583	− 1.72	1.0	6.5
**H6**	386.413	0	2.075	3	1031.416	83.314	11.847	− 3.425	− 0.85	1.0	7.0
**H7**	396.478	0	3.264	3	1137.026	92.005	11.244	− 3.312	− 0.77	1.0	6.5
**H8**	396.478	0	3.238	3	1143.692	90.455	11.336	− 3.466	− 0.917	1.0	6.5
**H9**	410.505	0	3.563	3	1194.624	93.175	11.135	− 3.488	− 0.862	1.0	6.5
**H10**	410.505	0	3.406	3	1148.371	94.491	11.07	− 3.17	− 0.599	1.0	6.5
**H11**	400.442	0	3.093	3	1092.485	89.859	11.429	− 3.349	− 0.752	1.0	6.5
**H12**	461.347	0	3.495	3	1136.077	91.969	11.403	− 3.439	− 0.723	1.0	6.5
**H13**	427.449	0	2.262	3	1154.785	69.373	12.711	− 5.044	− 1.858	1.0	7.5
**H14**	461.894	0	2.863	3	1181.117	77.794	12.25	− 4.654	− 1.299	1.0	7.5
**H15**	412.477	0	3.051	3	1160.063	89.311	11.907	− 3.34	− 0.915	1.0	7.25
**H16**	412.477	0	3.027	3	1123.234	93.623	11.593	− 2.874	− 0.549	1.0	7.25
**H17**	412.477	0	3.033	3	1159.589	89.485	11.807	− 3.362	− 0.926	1.0	7.25
**H18**	400.442	0	3.174	3	1101.044	90.007	11.432	− 3.408	− 0.777	1.0	6.5
**H19**	427.449	0	2.238	3	1158.399	68.166	12.751	− 5.165	− 1.94	1.0	7.5

### Anticancer potential

**Fig. 5:**
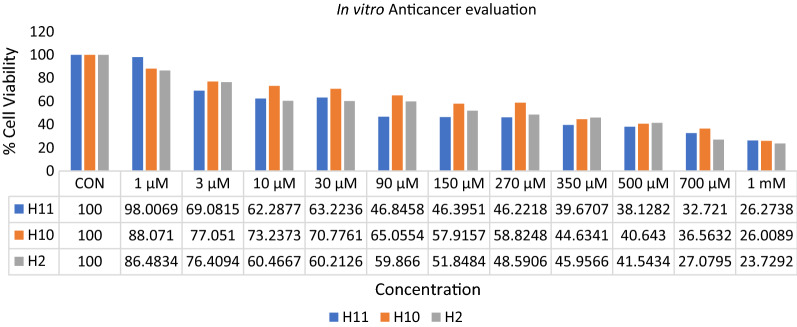
Anticancer evaluation results of compounds **H2, H10** and **H11** against DU-145 prostate cancer cell lines

Anticancer potential of three synthesized derivatives viz*.* **H2**, **H10** and **H11** were tested for their in vitro anticancer potential against prostate cancer cell line (DU-145) using MTT assay. The results of anticancer evaluation of all the screened derivatives were summarized in Fig. 5.

### Structure activity relationship (SAR)

**Fig. 6:**
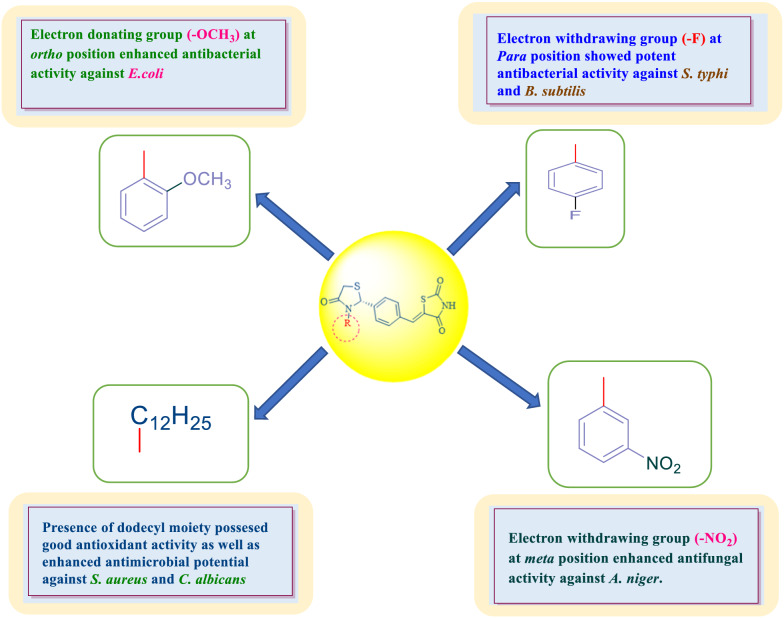
Structure activity relationship of synthesized compounds

From the results of antimicrobial, anticancer and antioxidant evaluation studies, the following SAR can be expressed (Fig. 6):

- Various substituent(s) present on aliphatic/aromatic amines used for the synthesis of the final analogues of 5-(-4-(3-(substituted aryl/alkyl)-4-oxothiazolidin-2-yl)benzylidene)thiazolidine-2,4-dione (**H1–H19**), have vital impact on the antimicrobial, anticancer and antioxidant potential of the synthesized molecules.
- Presence of electron donating group (–OCH_3_) at ortho position in molecule **H15** enhanced antibacterial activity against *E. coli.*
- Substitution of nitro (–NO_2_) group at meta position (electron withdrawing) in molecule **H13** enhanced antifungal activity against *A. niger*.
- Presence of (–F) group (electron withdrawing) at para position in molecule **H18** enhanced the antibacterial activity against *S. typhi* and *B. subtilis* whereas substitution of aliphatic group dodecyl in the derived molecule **H5**, enhanced the antioxidant activity and also exhibited better antimicrobial potential against *S. aureus* and *C. albicans*.

## Conclusion

A series of thiazolidin-2,4-dione clubbed with thiazolidin-4-one molecules was synthesized and then screened for its antimicrobial, anticancer and antioxidant potential. The molecules **H5**, **H13**, **H15** and **H18** exhibited moderate to good antimicrobial activity against various selected strains of microbial species with MIC ranging from 7.3 µM to 26.3 µM in the antimicrobial screening assay. In the antioxidant evaluation assay, the compound **H5** was found to be the best antioxidant molecule among the synthesized series with an IC_50_ value of 14.85 μg/mL. ADME studies revealed that all the compounds found to be drug-like to be orally active as all the parameters of the compounds was found within Lipinski’s rule of five. These derivatives can be used as lead structures for further modification/optimization into more potent antimicrobial, anticancer and antioxidant drug molecules with least toxicity.

## Experimental

The chemicals used in the synthetic work were either of AR or LR grade purchased from different vendors and were used as such without any further purification.

The melting point (m.p.) of the synthesized compounds was determined by using Stuart scientific SMP3 apparatus on open glass capillaries and reported uncorrect. The progress of every synthetic step was monitored using TLC (Thin layer chromatography) on precoated silica gel G plates. Bruker 12060280 (Software: OPUS 7.2.139.1294) spectrophotometer was used for recording infrared (IR, KBr, cm^−1^) by taking KBr pellets. ^1^H spectral determination was recorded on Bruker Avance III 400 NMR spectrometer by taking appropriate deuterated solvents and are expressed in parts per million (δ, ppm) downfield from internal standard tetramethylsilane. Waters Micromass Q-ToF Micro instrument was used for recording the mass spectra of the synthesized derivatives. Elemental analysis was recorded using CHNS analyzer.

## Synthetic steps of Scheme 1

- Step 1: Synthesis of thiazolidin-2,4-dione TZD (**INT-I**):Aqueous solution of thiourea (0.06 mol in 15 mL of water) was added dropwise to the aqueous solution of chloroacetic acid (0.06 mol in 15 mL of water) and stirred constantly till the formation of white precipitates. 6 mL of concentrated HCl was then added dropwise in the above mixture and then refluxing was carried for 10 h. Fine needle shaped crystals of TZD (**INT-I**) were formed on cooling which were filtered, dried and recrystallized from methanol [27].
- Step 2: Synthesis of (E)-4-((substitutedaryl/alkylimino)methyl)benzaldehyde (**1–19**):
  To a solution of terephthalaldehyde (0.01 mol) in ethanol (25 mL), various substituted amines/anilines in equimolar amount were added using acetic acid (glacial) as a catalyst and refluxing was done for 6–15 h. The reaction mixture was then allowed to cool to obtain solid mass which was further recrystallized from methanol to give intermediate Schiff’s bases **(1–19)** [37].
- Step 3: Synthesis of 4-(4-oxo-3-substitutedaryl/alkylthiazolidin-2-yl)benzaldehyde (**A1–A19**):
  The solution of 0.015 mol of thioglycolic acid in 20 ml of *N*,*N*-dimethylformamide (DMF) was added dropwise with constant stirring to the (0.01 mol) solution of Schiff’s base (**1–19**) prepared in DMF. A pinch of anhydrous ZnCl_2_ was added to the reaction mixture followed by refluxing for about 6–8 h. The reaction mixture was then cooled and poured on to crushed ice. The solid mass so obtained was filtered and washed with cold distilled water. The product after drying was recrystallized from methanol to obtain intermediate derivatives (**A1–A19**) [38].
- Step 4: Synthesis of various title compounds (**H1–H19**):
  To the solution of **INT-I** (0.01 mol) in DMF (10 mL), compounds **A1–A19** in DMF were added, followed by addition of 3 mL of piperidine (0.0188 mol). The contents of the flask was then stirred and refluxed for next 12–24 h. On completion of the reaction; the reaction mixture was then poured on ice followed by acidification with acetic acid (glacial) to obtain final crude compounds 5-(-4-(3-(substitutedaryl/alkyl)-4-oxothiazolidin-2-yl)benzylidene) thiazolidine-2,4-dione (**H1–H19**). The products after drying were recrystallized from methanol to obtain final pure compounds (**H1–H19**).

### In vitro antimicrobial evaluation

The synthesized molecules were screened for their antimicrobial potential employing serial tube dilution method [32] by comparing with marketed antibiotics; cefadroxil (antibacterial screening) fluconazole (antifungal screening). The evaluation was carried out using both Gram − ve {MTCC-3231 (*S. typhi*), MTCC-443 (*E. coli*)} and Gram + ve {MTCC-441 (*B. subtilis*), MTCC-3160 (*S. aureus*)} bacterial strains. The antifungal screening study was assessed using two fungal strains {MTCC-281 (*A. niger*), MTCC-227 (*C. albicans*)}. The antimicrobial screening was carried out by using Sabouraud dextrose broth I.P. (for fungi) or Nutrient broth double strength I.P. (for bacteria) [39] nutrient media. Dimethyl sulfoxide was used as diluting medium for the preparation of stock solutions of the reference and test molecules along with a control set of the same dilutions. Incubation of the samples at 37 ± 1 °C (24 h) for bacteria, at 37 ± 1 °C (48 h) for *C. albicans* and at 25 ± 1 °C (7 days) for *A. niger*, respectively. Results were recorded as MIC for the tested molecules that exhibited no observable growth of microorganisms in the test tube at the lowest possible concentration.

### In vitro antioxidant assay

The antioxidant potential of synthesized thiazolidine-2,4-dione molecules was analyzed using DPPH free radical scavenging assay [40]. Synthesized compounds were diluted to 25 μg/mL, 50 μg/mL, 75 μg/mL and 100 μg/mL concentration with methanol and kept in different test tube. To these test tubes equal quantity of 0.0039% DPPH in methanol was added followed by vigorous shaking. The test tubes containing the above mixture were then wrapped with silver foil paper and kept in a dark room for 30 min. Finally, a UV–Visible double beam spectrophotometer was used to measure the absorbance of the mixtures at 517 nm. The mean IC_50_ value of at least three observations is presented in the data.

### In vitro anticancer screening

The anticancer screening was done using MTT (3-(4,5-dimethylthiazol-2-yl)-2,5-diphenyltetrazolium bromide) assay. Firstly, cells of DU-145 were seeded (5 × 10^3^ cells/well) at clear 96-well plates. After 24 h, synthesized derivatives in a concentration of 1 µM, 3 µM, 10 µM, 30 µM, 90 µM, 150 µM, 270 µM, 350 µM, 500 µM, 700 µM and 1 mM were added to each well to expose the cell lines for further 24 h. After the treatment, the 0.5 mg/mL MTT reagent solution was added and the plates were incubated at 37 °C for 4 h. MTT solution was then removed by inverting the well plate and 150 μL of DMSO was then added to dissolve insoluble formazan crystals. The optical density at 570 nm was determined spectrophotometrically. The anticancer potential was expressed as the relative cell viability (%) relative to the untreated control cells [41].

## Data Availability

All data generated or analyzed during this study are included in this published article.

## Abbreviations

MIC: Minimum inhibitory concentration
FT-IR: Fourier transform infrared spectroscopy
IC_50_: Half maximal inhibitory concentration
μM: Micro molar
TLC: Thin layer chromatography
MTT: (3-(4,5-Dimethylthiazolyl-2)-2,5-diphenyltetrazolium bromide)
^1^H-NMR: Proton nuclear magnetic resonance spectroscopy
MS: Mass spectrometry
ADME: Absorption, distribution, metabolism and excretion
DPPH: 2,2-Diphenyl-1-picrylhydrazyl
MRSA: Multidrug resistant *Staphylococcus aureus*
VRE: Vancomycin-resistant *Enterococci*
